# Induced Pluripotent Stem Cell-Derived Conditioned Medium Promotes Endogenous Leukemia Inhibitory Factor to Attenuate Endotoxin-Induced Acute Lung Injury

**DOI:** 10.3390/ijms22115554

**Published:** 2021-05-24

**Authors:** Vincent Yi-Fong Su, Shih-Hwa Chiou, Wei-Chih Chen, Wen-Kuang Yu, Huai-Hsuan Wu, Hao Chen, Kuang-Yao Yang

**Affiliations:** 1 School of Medicine, Faculty of Medicine, National Yang Ming Chiao Tung University, Taipei 112, Taiwan; bsbipoke@hotmail.com (V.Y.-F.S.); shchiou@vghtpe.gov.tw (S.-H.C.); wcchen2@vghtpe.gov.tw (W.-C.C.); wkyu2@vghtpe.gov.tw (W.-K.Y.); 2 Department of Internal Medicine, Taipei City Hospital, Taipei City Government, Taipei 108, Taiwan; 3 Department of Medical Research and Education, Taipei Veterans General Hospital, Taipei 11217, Taiwan; 4 Department of Pharmacology, School of Medicine and Institute of Pharmacology, College of Medicine, National Yang Ming Chiao Tung University, Taipei 112, Taiwan; 5 Department of Chest Medicine, Taipei Veterans General Hospital, Taipei 11217, Taiwan; purplewings0401@gmail.com (H.-H.W.); asura811218@gmail.com (H.C.); 6 School of Medicine, Institute of Emergency and Critical Care Medicine, National Yang Ming Chiao Tung University, Taipei 112, Taiwan; 7 Cancer Progression Research Center, National Yang Ming Chiao Tung University, Taipei 112, Taiwan

**Keywords:** induced pluripotent stem cell, acute lung injury, LIF, neutrophil transendothelial migration

## Abstract

The conditioned medium of induced pluripotent stem cells (iPSC-CM) can attenuate neutrophil recruitment and endothelial leakage of lipopolysaccharide (LPS)-induced acute lung injury (ALI). Therefore, we investigated the mechanisms by which iPSC-CM regulate the interaction between neutrophils and the endothelium in ALI. Murine iPSCs (miPSCs) were delivered intravenously to male C57BL/6 mice (8–12 weeks old) 4 h after intratracheal LPS injection. A miPSC-derived conditioned medium (miPSC-CM) was delivered intravenously to mice after intratracheal LPS injection. DMSO-induced HL-60 cells (D-HL-60, neutrophil-like cells) and human umbilical vein endothelial cells (HUVECs) were used as in vitro models to assess the interaction of neutrophils and endothelial cells. miPSC-CM diminished the histopathological changes in the lungs and the neutrophil count in bronchoalveolar lavage fluids of ALI mice. miPSC-CM attenuated the expression of adhesion molecules in the lungs of ALI mice. Human iPSC conditioned medium (hiPSC-CM) reduced the expression of adhesion molecules in a HUVEC and D-HL-60 co-culture after LPS stimulation, which decreased the transendothelial migration (TEM) of D-HL-60. A human angiogenesis factors protein array revealed that leukemia inhibitory factor (LIF) was not detected in the absence of D-HL-60 and hiPSC-CM groups. hiPSC-CM significantly promoted the production of endogenous LIF in in vitro models. Administration of an anti-LIF antibody not only reversed the effect of iPSC-CM in ALI mice, but also blocked the effect of iPSC-CM on neutrophils TEM in in vitro models. However, a controlled IgG had no such effect. Our study demonstrated that iPSC-CM promoted endogenous LIF to inhibit neutrophils TEM and attenuate the severity of sepsis-induced ALI.

## 1. Introduction

Sepsis is characterized by an excessive inflammatory response to infectious pathogens. Acute lung injury (ALI) is a common respiratory complication of severe sepsis that is characterized by acute and severe hypoxia. In its most severe form, acute respiratory distress syndrome (ARDS) can occur and result in substantial respiratory failure with high mortality. ALI/ARDS is characterized by the diffuse infiltration of neutrophils to the alveolar space, loss of alveolar–capillary membrane integrity, and increased permeability of pulmonary capillary endothelial cells [1]. To date, the management of ARDS remains confined to supportive care, including the use of a neuromuscular blockade, lung-protective mechanical ventilation strategies, prone positioning, and fluid-conservative strategies. Stem cells have a naturally regenerative and anti-inflammatory effect; hence, they have great potential for future therapeutic uses in tissue regeneration and repair. Accumulating studies have shown that stem cell therapy is becoming one of the emerging treatment strategies for ALI/ARDS. Recently, research about stem cell-based therapies for ALI have been published; our previous study showed that mesenchymal stem cells (MSCs) attenuate endotoxin-induced ALI by inducing neutrophil apoptosis, which is associated with inhibition of the NF-κB pathway [2]. Induced pluripotent stem cells (iPSCs) are a novel type of pluripotent stem cell and are generated from differentiated somatic cells by reprogramming certain transcription factors [3,4]. Recently, as the concern of iPSC-related teratoma formation has increased, several studies have focused on iPSC-derived MSC, secretory exosome, or conditioned medium (iPSC-CM), but there are very few studies that have explored the mediators induced by the conditioned medium. Therefore, we investigated the regulatory mediators enhanced by iPSC-CM to attenuate endotoxin-induced ALI.

Our previous work [5,6,7,8] showed that iPSC-CM reduces the severity of sepsis-induced ALI and improves associated pathological changes. We found that iPSC-CM reduced nuclear factor (NF)-κB activity [5] and neutrophil chemotaxis [6] by reducing the expression of TREM-1 and p38 mitogen-activated protein kinase signaling [7] in sepsis-induced ALI. Furthermore, iPSC-CM reduce the permeability of endothelial cells by reversing the LPS-induced suppression of VE-cadherin in pulmonary endothelium and down-regulating the expression of pFAK-Tyr397 and Snail via the tissue inhibitor of metalloproteinase-1 in LPS-induced ALI [8]. However, the mechanism by which iPSC-CM is able to regulate the interaction between neutrophils and endothelium in sepsis-induced ALI remains unclear.

There is accumulating evidence demonstrating that the adhesion molecules expressed on the endothelium are important regulators of acute inflammatory responses. Both intercellular adhesion molecule-1 (ICAM-1) and vascular cell adhesion molecule-1 (VCAM-1) have been implicated in the migration of neutrophils during sepsis-induced ALI [9]. In the lungs, ICAM-1 is widely expressed in human tissues, including the vascular endothelium and inflammatory cells, and can be induced by pro-inflammatory stimuli [10]. The very late antigen-4 (VLA-4), which is a member of the integrin family, is expressed on circulating leukocytes and binds to VCAM-1 expressed by endothelial cells. ICAM-1 is a ligand for lymphocyte-function-associated antigen-1 (LFA-1), which is an integrin found on leukocytes. Clearly, the interactions of both VCAM-1/VLA-4 and ICAM-1/LFA-1 appear to be critical for the transendothelial migration (TEM) of neutrophils in ALI and ARDS [11,12,13]. We have previously demonstrated that iPSC-CM attenuates NF-κB activity, neutrophil chemotaxis, and endothelial leakage of sepsis-induced ALI. Therefore, this study investigated how iPSC-CM therapy affects neutrophil TEM in LPS-induced ALI.

## 2. Results

### 2.1. Effects of miPSC-CM on the Histopathology and Neutrophil Accumulation in the Lung in LPS-Induced ALI Mice

**Figure 1:**
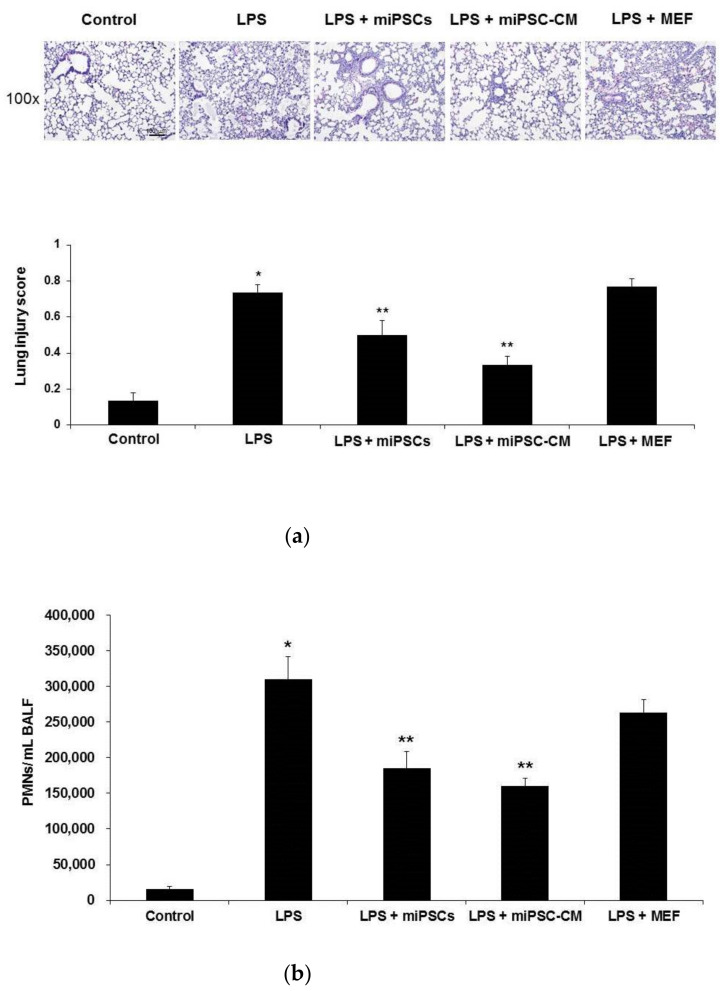
Administration of murine-induced pluripotent stem cells (miPSCs) or conditioned medium of miPSCs (miPSC-CM) improved the histological features of mice with lipopolysaccharide (LPS)-induced acute lung injury (ALI) and prevented the accumulation of neutrophils after LPS-induced ALI. (**a**) Hematoxylin and eosin staining of lung sections (original magnification ×100) demonstrated the attenuation of lung injury in mice receiving miPSCs (LPS + miPSCs) or miPSC-CM (LPS + miPSC-CM) 24 h after LPS-induced ALI (LPS). Control mice injected with intratracheal phosphate-buffered saline (PBS) showed minimal histopathological abnormalities. ALI mice that received miPSCs or miPSC-CM had significantly lower histopathological lung injury scores compared to ALI mice. The lung injury score represents the average result from two independent readers. Data are the mean ± standard deviation (SD). Mice that received mouse embryonic fibroblasts (MEFs; LPS + MEFs) showed no improvement in the severity of LPS-induced ALI compared to untreated mice (LPS) at 24 h. (**b**) Bronchoalveolar lavage fluid (BALF) showed a marked accumulation of polymorphonuclear neutrophils (PMNs) in the lungs of LPS-induced ALI mice (LPS). Mice treated with miPSCs or miPSC-CM had significantly lower numbers of BALF PMNs. Data are the mean ± SD. * *p* < 0.05, compared to control mice; ** *p* < 0.05, compared to LPS-induced ALI mice; *n* = 6 per group.

Intratracheal injection of the endotoxin resulted in extensive ALI, as evidenced histopathologically by lung edema, alveolar spaces filled with mononuclear/neutrophilic infiltrates, and the thickening of the alveolar walls and interstitium. Compared to ALI mice, histopathological changes of lungs in LPS-induced ALI mice could be significantly reduced by miPSC or miPSC-CM treatment (Figure 1a). In contrast, the addition of MEF did not change the severity of LPS-induced ALI. BALF analyses revealed a marked accumulation of polymorphonuclear neutrophils (PMNs) in the lungs of LPS-induced ALI mice, whereas miPSC or miPSC-CM-treated mice had significantly reduced numbers of PMN (Figure 1b).

### 2.2. miPSC-CM Reduced ICAM-1/LFA-1 and VCAM-1/VLA-4 Expression in Lung Tissues Following LPS-Induced ALI

**Figure 2:**
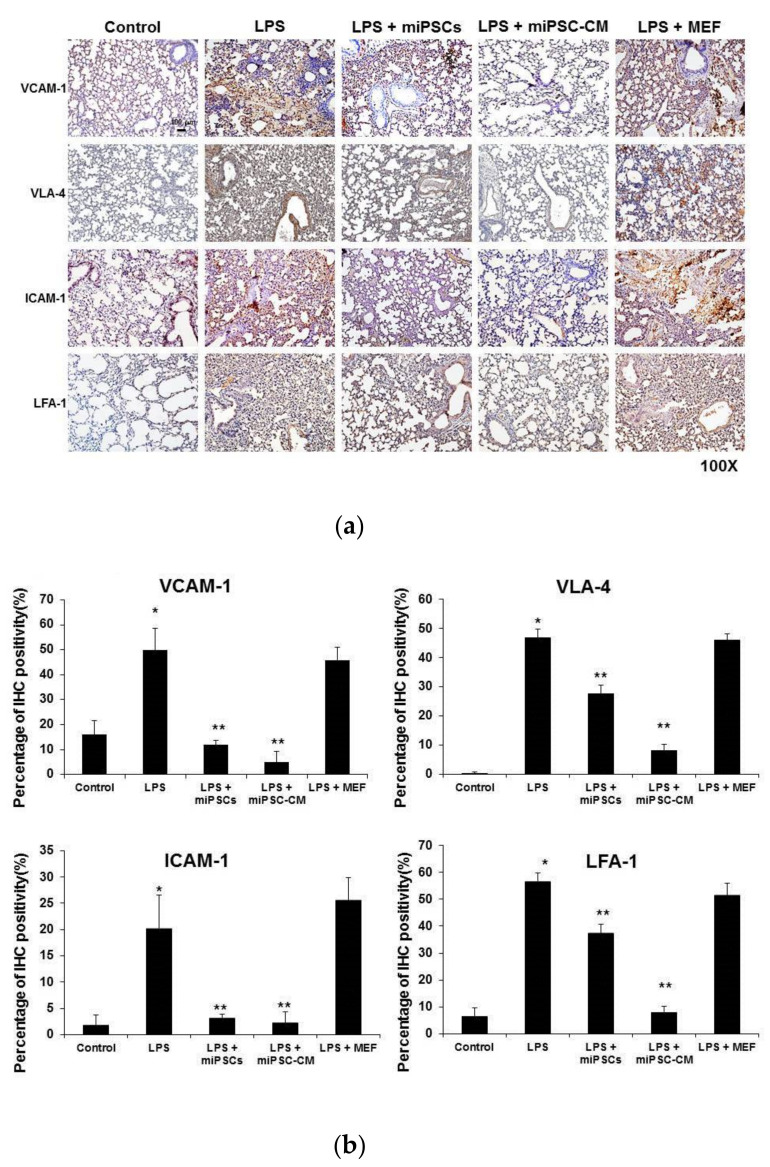
Administration of LPS increased the expression of adhesion molecules (including VCAM-1, VLA-4, ICAM-1, and LFA-1) in lung tissue. Administration of miPSCs or miPSC-CM reduced adhesion molecules in mice with LPS-induced ALI. (**a**,**b**) Immunohistochemistry (IHC) staining of lung sections show that VCAM-1 expression significantly increased following the administration of LPS compared to control mice. In contrast, the expression of VCAM-1 in ALI mice was reduced by intravenous delivery of miPSCs or miPSC-CM. Similarly, the expression of VLA-4, ICAM-1, and LFA-1 in the lung was increased by LPS stimulation and was inhibited by administering miPSCs or miPSC-CM. Data are presented as the mean ± SD. * *p* < 0.05, compared to control mice; ** *p* < 0.05, compared to LPS-induced ALI mice; *n* = 6 per group.

Compared to the control (wild type C57BL/6 mice), immunohistochemical staining of lung tissue showed that the expression levels of VCAM-1 (the most important counter-receptor of VLA-4) and VLA-4 significantly increased 24 h after LPS-induced ALI (VCAM-1: 49.9% vs. 16%; VLA-4: 46.8% vs. 0.3%, respectively; *p* < 0.05) (Figure 2a,b). LPS also induced the expression of ICAM-1 (the most important counter-receptor of LFA-1) and LFA-1 in the lung (ICAM-1: 20.3% vs. 1.7%; LFA-1: 56.8% vs. 6.7%, respectively; *p* < 0.05). However, intravenous injection of miPSCs or miPSC-CM significantly decreased the expression of VCAM-1/VLA-4 and ICAM-1/LFA-1 interactions in the lung of LPS-induced ALI mice.

**Figure 3:**
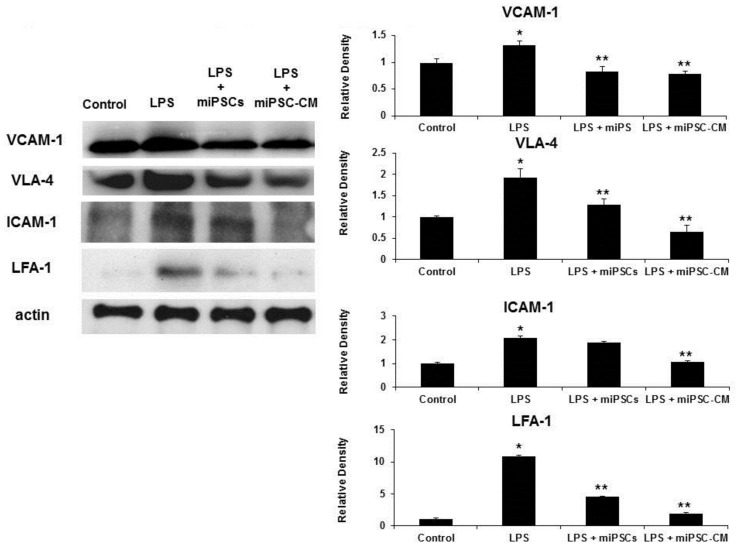
Western blot assays confirmed that LPS increased the expression of VCAM-1, VLA-4, ICAM-1, and LFA-1 in lung tissue. Administration of miPSCs or miPSC-CM reduced the expression of VCAM-1 and VLA-4 in LPS-induced ALI mice. However, the expression of ICAM-1 in LPS-induced ALI mice could not be reduced by miPSCs. Data are presented as the mean ± SD. * *p* < 0.05, compared to control mice; ** *p* < 0.05, compared to LPS-induced ALI mice; *n* = 6 per group.

Western blotting of whole lung extracts confirmed that the expression of VCAM-1/VLA-4 and ICAM-1/LFA-1 interactions increased following the administration of LPS to wild type mice. The administration of miPSC-CM, following LPS-induced ALI, reduced the expression of VCAM-1/VLA-4 and ICAM-1/LFA-1 interactions (Figure 3).

### 2.3. Effects of hiPSC-CM on Human Neutrophil–Endothelial Cell Interactions

**Figure 4:**
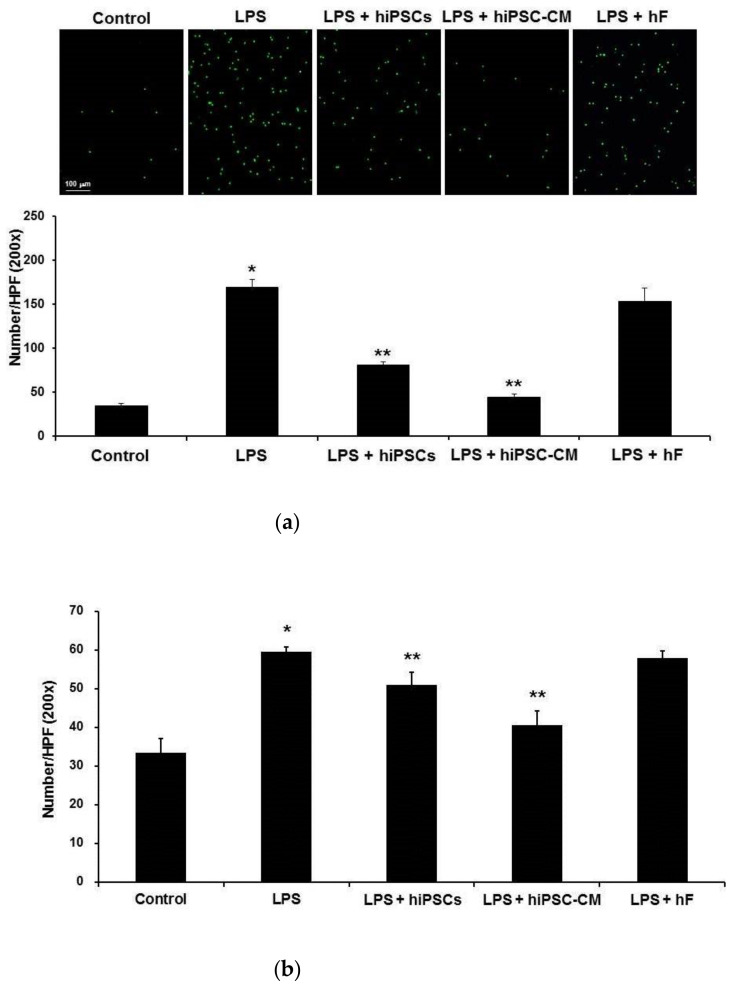

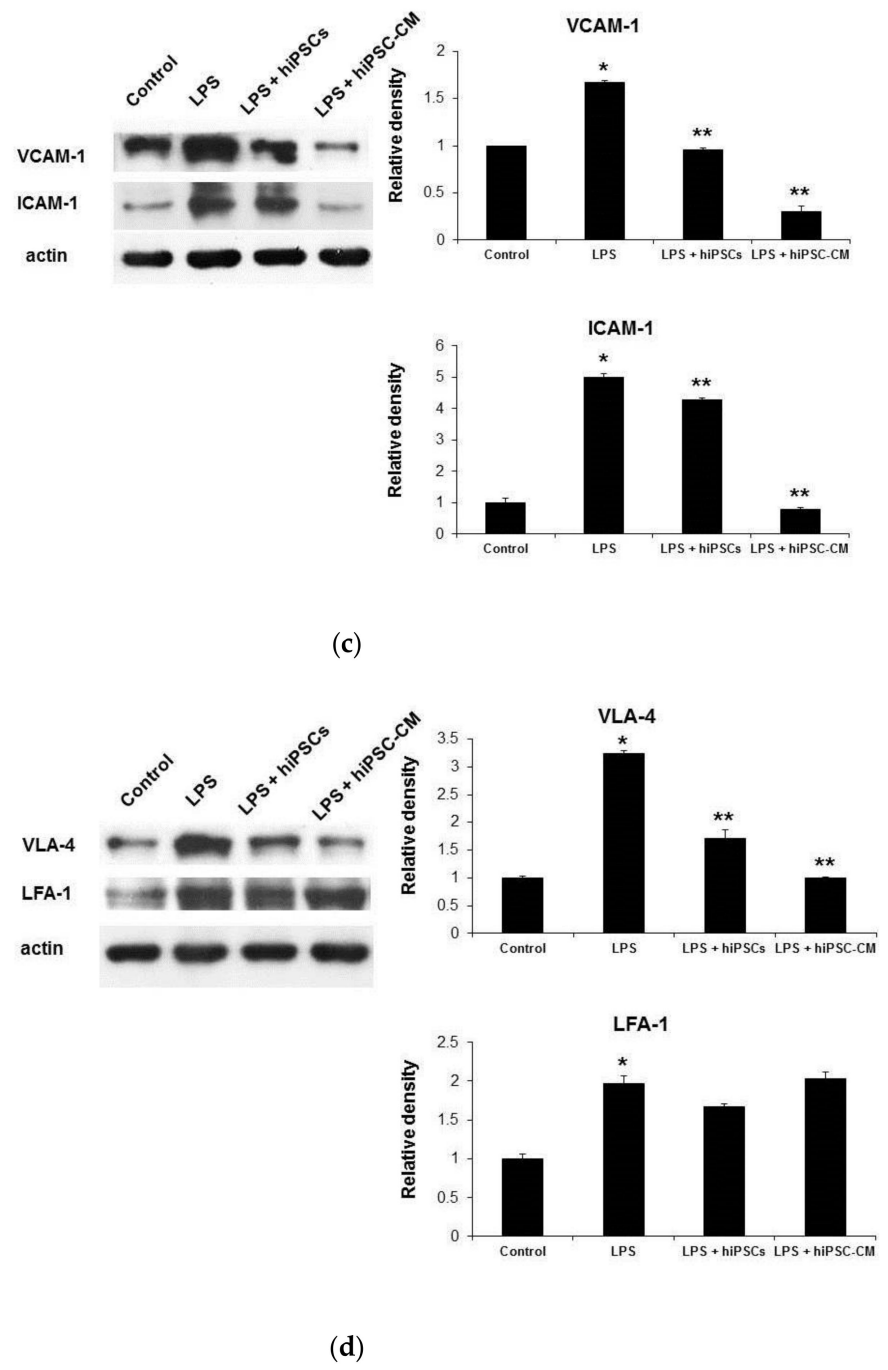
Administration of hiPSCs or hiPSC-CM reduced human neutrophils (D-HL-60 cells) adhering to human endothelial cells (HUVECs) and inhibited the TEM of neutrophils induced by LPS. Administration of hiPSCs or hiPSC-CM reduced the VCAM-1 expression induced by LPS in HUVEC and VLA-4 induced by LPS in D-HL-60 cells. (**a**) The D-HL-60 cell/HUVEC adhesion assay showed that D-HL-60 cells adhering to HUVECs significantly increased after administering LPS compared to the control. In contrast, the D-HL-60 cell/HUVEC adhesion induced by LPS was reduced by the intravenous delivery of miPSCs or miPSC-CM. (**b**) Similarly, the D-HL-60 cell/HUVEC transmigration assay showed that D-HL-60 cell TEM significantly increased after administering LPS and was reduced by the intravenous delivery of miPSCs or miPSC-CM. (**c**) Western blot assays showed that the expression of VCAM-1 and ICAM-1 in HUVECs significantly increased after administering LPS and was reduced by the intravenous delivery of miPSCs or miPSC-CM. (**d**) Western blot assays showed that the expression of VLA-4 in D-HL-60 cells significantly increased after administering LPS and was reduced by the intravenous delivery of miPSCs or miPSC-CM. However, the increased expression of LFA-1 in D-HL-60 cells induced by LPS was not reduced by miPSCs or miPSC-CM. Data are presented as the mean ± SD. * *p* < 0.05, compared to control mice; ** *p* < 0.05, compared to LPS-induced ALI mice; *n* = 6 per group.

We investigated the effects of hiPSCs on human neutrophil (D-HL-60 cell)–endothelial cell (HUVEC) interactions using the in vitro neutrophil–endothelial cell adhesion model and the neutrophil TEM model. In in vitro neutrophil–endothelial cell adhesion model, the number of neutrophils that adhered to cultured human endothelial cells significantly increased following the administration of LPS. Compared to LPS administration alone, administration of hiPSCs or hiPSC-CM led to a significant decrease in the number of neutrophils that adhered to cultured human endothelial cells (Figure 4a). In in vitro neutrophil TEM model, the number of TEM neutrophils significantly increased following the administration of LPS. Compared to LPS administration alone, administration of hiPSCs or hiPSC-CM significantly decreased the number of TEM neutrophils (Figure 4b).

Western blot analysis showed that LPS caused adhesion molecule (VCAM-1 and ICAM-1) levels to increase significantly in HUVECs. Elevated VCAM-1 and ICAM-1 was also reduced by administering hiPSCs or hiPSC-CM (Figure 4c). Western blot analysis showed that LPS caused adhesion molecule (VLA-4 and LFA-1) levels to increase significantly in D-HL-60 cells. Only elevated VLA-4 was reduced by administering hiPSCs or hiPSC-CM. hiPSCs or hiPSC-CM did not reduce LFA-1 levels in D-HL-60 cells treated with LPS (Figure 4d).

### 2.4. hiPSC-CM Promoted Endogenous Leukemia Inhibitory Factor (LIF) Secretion to Decrease Neutrophils Transendothelial Migration (TEM) In Vitro

**Figure 5:**
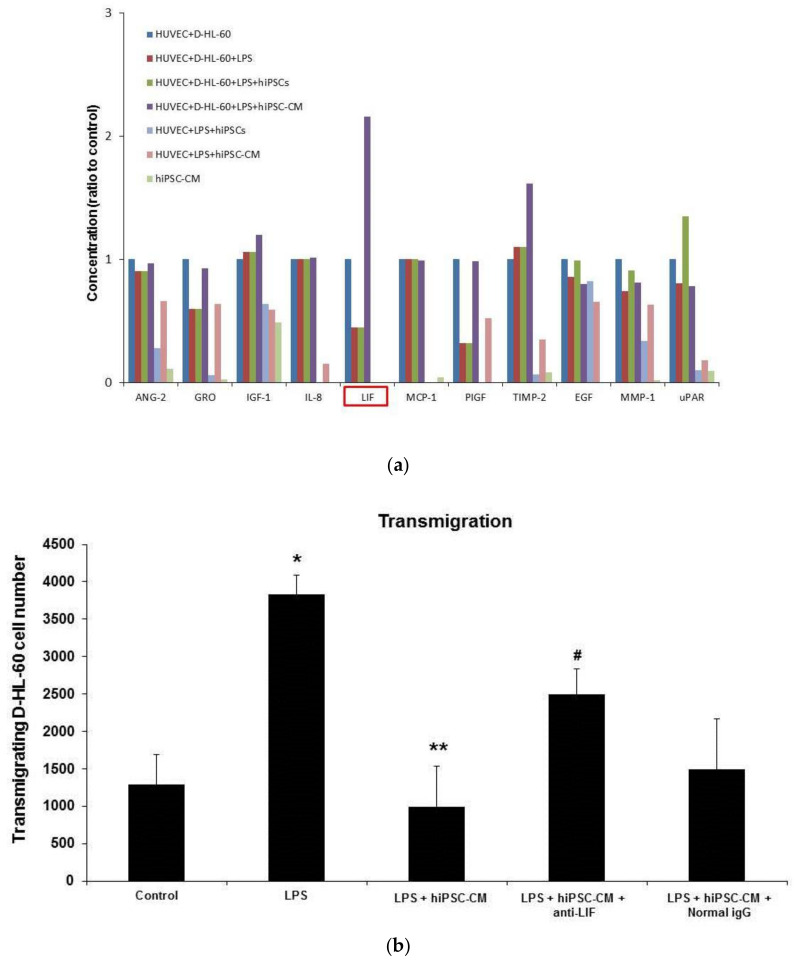
In vitro, iPSC-CM reduced D-HL-60 cell/HUVEC transmigration by inducing endogenous LIF from D-HL-60 cells. (**a**) The human angiogenesis factors protein array confirmed that there was no detectable LIF in hiPSC-CM. Endogenous LIF from D-HL-60 cells was induced after administering hiPSC-CM. (**b**) The D-HL-60 cell/HUVEC transmigration assay showed that administering LPS increased the D-HL-60 cell TEM, which was reduced by hiPSC-CM. Stimulation of LPS on the D-HL-60 cell TEM was significantly reduced by hiPSC-CM. Administration of the anti-LIF antibody inhibited the effect of hiPSC-CM on D-HL-60 cell TEM. Data are presented as the mean ± SD. * *p* < 0.05, compared to control mice; ** *p* < 0.05, compared to LPS-induced ALI mice; ^#^ *p* < 0.05, compared to LPS-induced ALI mice that received miPSC-CM; *n* = 6 per group.

A human angiogenesis factors protein array was used to detect the concentrations of angiogenic factors in different culture groups. The human angiogenesis array showed that the level of LIF in the HUVEC/D-HL-60 cell co-culture was reduced by LPS and restored by hiPSC-CM (Figure 5a). Importantly, the human angiogenesis factors protein array confirmed that there was no detectable LIF in hiPSC-CM. LIF levels were also not detectable in HUVEC + LPS + hiPSCs and HUVEC + LPS + hiPSC-CM groups.

An in vitro neutrophil TEM assay was used to confirm the important role of endogenous LIF. The number of D-HL-60 cells TEM was significantly increased by LPS administration, but the effect of LPS stimulation was significantly reduced by hiPSC-CM. Additionally, the effect of hiPSC-CM was blocked by administering the anti-LIF antibody. Normal IgG did not reduce the effect of hiPSC-CM (Figure 5b).

### 2.5. Anti-LIF Antibody Treatment Reversed the Effect of miPSC-CM on the Expression of VCAM-1 and VLA-4 and Pathological Changes in ALI Mice

**Figure 6:**
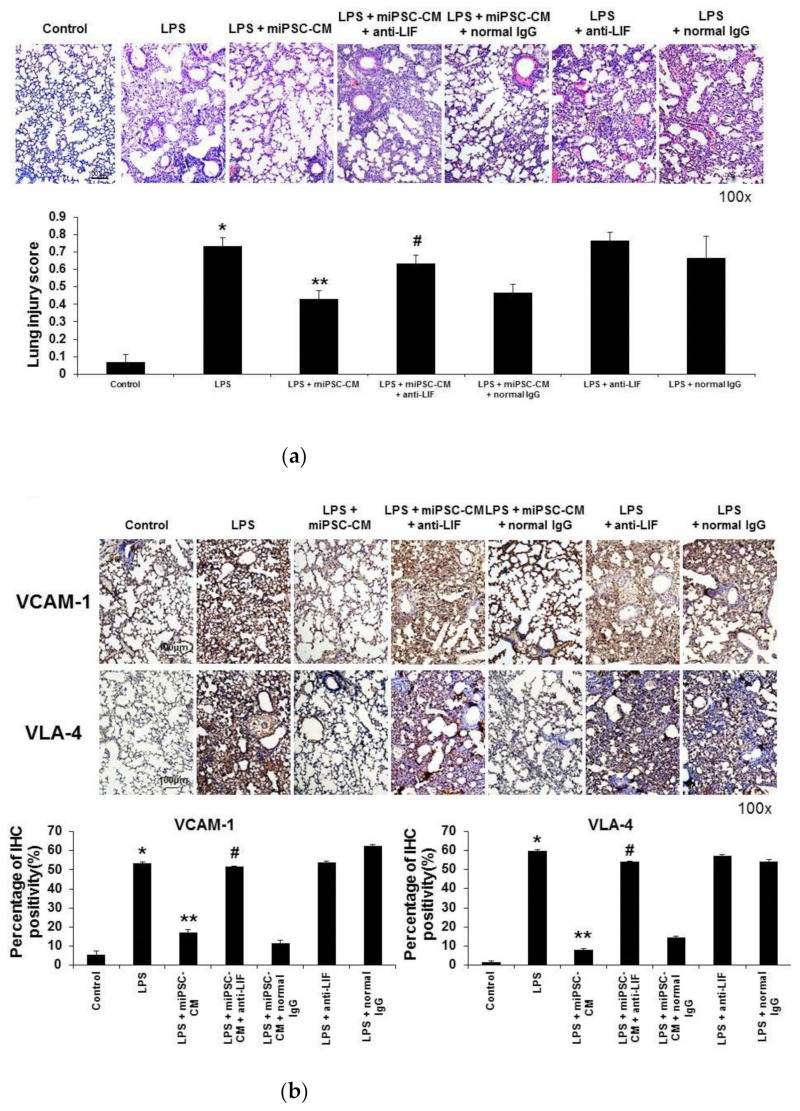

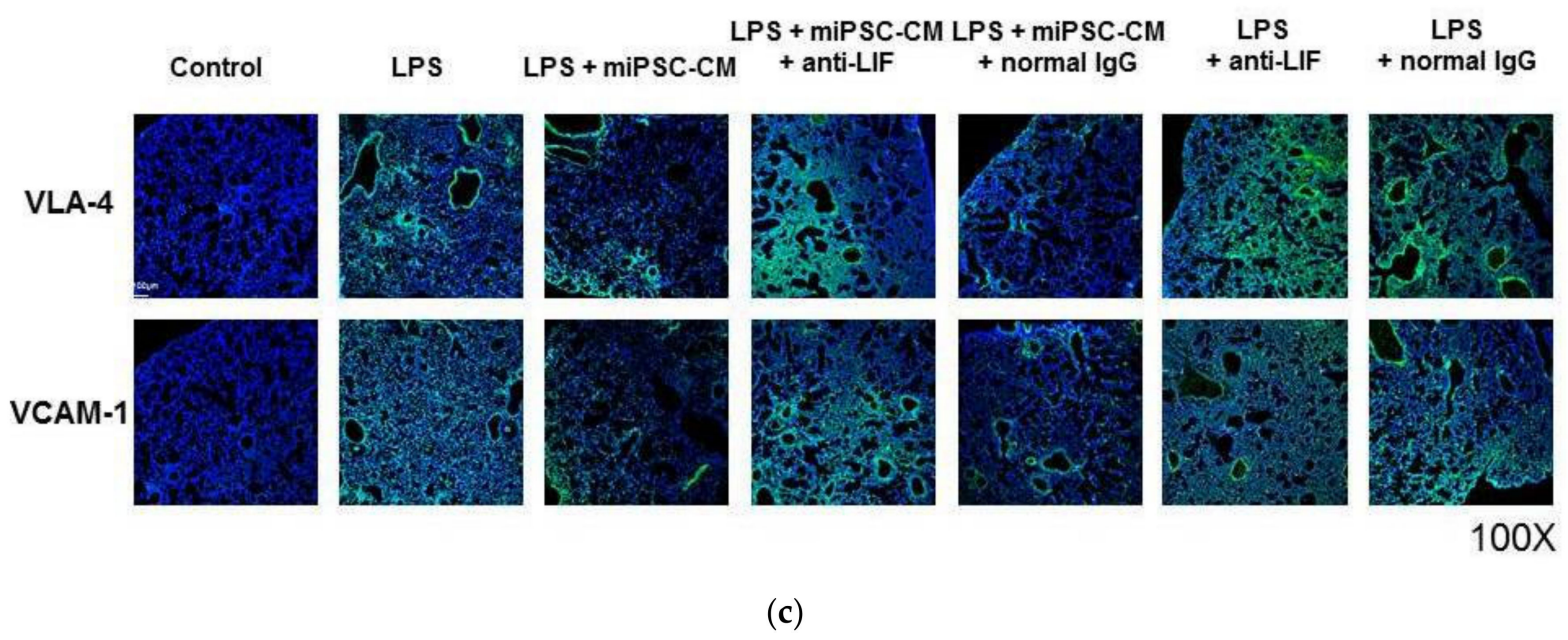
Adding the anti-LIF antibody reduced the beneficial effect of miPSC-CM on the severity of ALI and the TEM of neutrophils in LPS-induced ALI. In contrast, administering normal IgG had no beneficial effect on miPSC-CM in LPS-induced ALI. (**a**) Hematoxylin and eosin staining of lung sections (original magnification ×100) showed that the co-administration of anti-LIF antibody (LPS + miPSC-CM + anti-LIF) inhibited the beneficial effect of miPSC-CM on the pathological changes of lung injury in LPS-induced ALI mice. In contrast, adding normal IgG did not change the severity of lung injury in LPS-induced ALI mice with or without miPSC-CM treatment. Similarly, immunohistochemistry (IHC) staining of lung sections showed that adding the anti-LIF antibody inhibited the reduction effect of miPSC-CM on the expression of adhesion molecules of neutrophil TEM: VCAM-1 (**b**) and VLA-4 in LPS-induced ALI. (**c**) Immunofluorescence staining produced the same results as IHC. Data are presented as the mean ± SD. * *p* < 0.05, compared to control mice; ** *p* < 0.05, compared to LPS-induced ALI mice; ^#^ *p* < 0.05, compared to LPS-induced ALI mice received miPSC-CM; *n* = 6 per group.

An in vivo animal study was conducted to evaluate the robustness of the results in vitro (the HUVEC/D-HL-60 cells co-culture). Compared to ALI mice, the pathological change to the lungs of LPS-induced ALI mice was significantly reduced by miPSC-CM treatment. The addition of the anti-LIF antibody reduced the beneficial effects of miPSC-CM on the severity of LPS-induced ALI. In contrast, normal IgG did not change the severity of LPS-induced ALI treated by administering miPSC-CM (Figure 6a).

IHC confirmed that the addition of the anti-LIF antibody reduced the beneficial effects of miPSC-CM on the expression of VCAM-1 and VLA-4 in LPS-induced ALI. Normal IgG did not change the effects of miPSC-CM on the expression of VCAM-1 and VLA-4 in LPS-induced ALI. In contrast, the addition of the anti-LIF antibody did not change the expression of VCAM-1 and VLA-4 in LPS-induced ALI (Figure 6b). Immunofluorescence staining of VCAM-1 and VLA-4 revealed the same finding as IHC staining (Figure 6c).

## 3. Discussion

The results of this study indicated that iPSCs can regulate neutrophil TEM in LPS-induced ALI. Current clinical management strategies are respiratory support and restricted fluid input, and there is no suggested pharmacological treatment. Stem cell therapy is a non-invasive treatment that has been widely used in basic research and clinical studies on ARDS. Stem cells have also been noted to possess the ability to impart profound immunomodulatory effects in vivo. Our previous research showed that iPSC-CM reduces NF-κB activity [5] and neutrophil chemotaxis [6] by inhibiting the expression of triggering receptors expressed on myeloid cells 1 and p38 mitogen-activated protein kinase signaling [7] in sepsis-induced ALI. iPSC-CM also reduces the permeability of endothelial cells by restoring the sepsis-induced suppression of VE-cadherin in the pulmonary endothelium via a tissue inhibitor of metalloproteinase-1 in LPS-induced ALI [8]. Consequently, we hypothesized that iPSC-CM attenuates the severity of LPS-induced ALI via the neutrophil TEM pathway. Administration of iPSC-CM showed effects similar to those of iPSCs; thus, iPSC-CM contained more paracrine mediators than iPSCs. The decrease in pulmonary TEM was greater in the iPSC-CM group compared to the iPSCs group. Similarly, our previous studies indicated that a significantly greater reduction of NF-κB activity [5], neutrophil chemotaxis [6], and TREM-1 and p38 mitogen-activated protein kinase activity [7] and pulmonary endothelium leakage [8] in sepsis-induced ALI was observed in the iPSC-CM group versus the iPSCs group.

Interactions between VLA-4 and VCAM-1 play an important role in the adhesion of lymphocytes [14,15], monocytes [16], eosinophils, basophils [17], and neutrophils [18,19,20]. Thus, there is evidence that the migration of neutrophils across the endothelium is mediated by the adhesion molecules of neutrophils to endothelial cells through the interaction of VLA-4 and VCAM-1. LIF is a cytokine that belongs to the interleukin-6 cytokine family, and it plays an important role in multiple biological processes, including inducing neutrophil differentiation, modulating inflammatory responses, and stem cell self-renewal. LIF is expressed by a variety of cell types in vitro, including fibroblasts, immune cells, and endothelial cells, as well as by a number of tissues in adults [21,22]. LIF plays an important role in anti-inflammation and inhibiting the growth of leukemia cells in LPS-induced ALI mice [23]. In humans, LIF expression is induced under many conditions and is regulated by many different transcription factors. Circulating LIF levels are elevated in patients with sepsis and septic shock [24], with levels being correlated with the severity of disease [25]. In the animal model, administering LIF attenuates the severity of sepsis and septic shock induced by live *Escherichia coli* infection [26]. Furthermore, intratracheal injection of LIF reduces the recruitment of neutrophils and the acute inflammatory response in LPS-induced ALI mice [23]. Therefore, endogenous LIF attenuates sepsis and septic shock and downregulates tumor necrosis factor-α synthesis and release in LPS-induced sepsis mice [27].

The human angiogenesis array in our study indicated that the production of LIF in the HUVEC/D-HL-60 cells co-culture was induced by hiPSC-CM. Importantly, LIF levels could not be detected in the absence of D-HL-60, suggesting that endogenous LIF is synthesized by D-HL-60 stimulated by iPSC-CM. Based on our previous observation that iPSCs reduce the pathologic severity of LPS-induced ALI [5,6,7,8], we hypothesized that the iPSCs can attenuate the TEM in LPS-induced ALI. An anti-LIF antibody was used to confirm the key role of LIF in attenuating the TEM of neutrophils in LPS-induced ALI treated with iPSC-CM. Furthermore, the administration of the anti-LIF antibody reduced the effect of iPSC-CM on the TEM of neutrophils both in vitro and in vivo.

However, this study has some limitations. First, our results do not represent all of the effects of iPSCs on neutrophil recruitment during LPS-induced ALI. In the future, additional mechanisms related to iPSCs to ameliorate ALI must be explored, including the regulation of neutrophil–endothelial cell interactions. Previous studies have demonstrated that circulating LIF levels are correlated with the severity of sepsis in humans [24,25]. In the animal model, administering LIF reduces the severity of sepsis and recruitment of neutrophils in bacterial infections [23,26]. Furthermore, endogenous LIF attenuates acute inflammatory responses and downregulates TNFα synthesis in LPS-induced sepsis mice [27]. Our research demonstrated that hiPSC-CM induces LIF expression to increase human neutrophils (in a D-HL-60 cell/HUVEC co-culture model), while administering the anti-LIF antibody inhibited the beneficial effects of iPSC on neutrophil TEM, with these results being confirmed in both in vivo and in vitro models. However, more studies are needed to explore the mechanisms by which iPSCs regulate endogenous LIF in LPS-induced ALI.

In conclusion, this study demonstrated a novel mechanism of iPSC-CM in attenuating neutrophil recruitment in endotoxin-induced ALI, by inhibiting the TEM of neutrophils. Furthermore, this effect was mediated via iPSC-CM promoting endogenous LIF secretion.

## 4. Methods

### 4.1. Experimental Animals

Male C57BL/6 mice of 8–12 weeks of age were purchased from the National Experimental Animal Center (Taipei, Taiwan). The mice were maintained at the Laboratory Animal Center of Taipei Veterans General Hospital (Taipei, Taiwan). They were kept under a 12 h light/dark cycle and had access to food and water ad libitum. All experiments were conducted in accordance with the Institutional Animal Care and Use Committee-approved protocols (TVGH IACUC No.2013-192, 01/03/2014). The study was conducted according to the guidelines of the Declaration of Helsinki. This study was not human subject research and was exempt from review by the Institutional Review Board.

### 4.2. Generation and Culture of iPSCs

iPSCs were obtained from Dr. Shih-Hwa Chiou’s laboratory (National Yang Ming Chiao Tung University, Taipei, Taiwan). Our previous work [5,6,7,8] demonstrated the pluripotency of iPSCs and the ability of iPSCs to attenuate ALI. Thus, murine iPSCs (miPSCs) were generated from mouse embryonic fibroblasts (MEFs) recovered from C57BL/6 mice. Human skin fibroblasts (hFs) were used to generate human iPSCs (hiPSCs). Retroviral vectors encoding three transcription factors (Oct4, Sox2, and Klf4) were used to induce iPSC reprogramming [3,4,5]. Characteristics of hiPSCs cultured in a serum-free/feeder-free system were comparable to those of miPSCs cultured in the conventional MEF feeder system. miPSCs and hiPSCs demonstrated multilineage differentiation potential. Importantly, LIF was not added in the preparation of iPSCs or iPSC-CM.

### 4.3. Experimental Design

An established murine model of LPS-induced ALI was used [2,5,6,7,8]. In brief, mice were subjected to anesthesia before receiving an intratracheal delivery of LPS from *Escherichia coli* (0111:B4; Sigma-Aldrich, St. Louis, MO, USA) at a dose of 5 mg/kg in 50 µL phosphate-buffered saline (PBS). Four hours later, mice received an intravenous tail vein injection of 300 µL PBS, 300 µL PBS with miPSCs (2 × 10^6^ cells), or 300 µL miPSC-CM to establish ALI mice, miPSC-treated ALI mice, or miPSC-CM-treated ALI mice, respectively. For the anti-LIF group and normal IgG group, 25 ng per mouse of anti-LIF (AF449, R&D, Minneapolis, MN, USA), or normal IgG (AB-108-C, R&D, Minneapolis, MN, USA) was given by intravenous tail vein injection at the same time as miPSC or miPSC-CM treatment. Control mice received an intratracheal delivery of 50 µL PBS. Twenty-four hours later, all mice were sacrificed. The tracheas were immediately exposed and cannulated with an 18-gauge blunt needle to lavage the lungs with 0.5 mL PBS three times. For each mouse, a total infusion of 1.5 mL PBS resulted in a final recovery of 1 mL bronchoalveolar lavage fluid (BALF). Cells in the BALF were counted with a hemocytometer. Neutrophil counts were calculated by cytospin with a Wright’s stain. Tissue samples were also collected from each mouse for histology and immunohistochemistry (IHC) assays.

### 4.4. Culture of Human Umbilical Vein Endothelial Cells (HUVECs)

Human umbilical vein endothelial cells (HUVECs) were employed as a model of neutrophil TEM. Lifeline Cell Technology was used to harvest HUVECs [28,29]. The cells were grown in VascuLife^®^ EnGS medium (Lifeline Cell Technology, Frederick, MD, USA; LS-1019; including 5 ng/mL rhEGF, 0.2% EnGS, 0.75 U/mL, 2% FBS, 10 mM L-glutamine, 1 μg/mL hydrocortisone, and 50 μg/mL ascorbic acid). The cell cultures were incubated in room air with 5% CO_2_ at 37 °C and 95% humidity and were expanded by brief trypsinization with 0.25% trypsin in PBS containing 0.025% ethylenediaminetetraacetic acid (EDTA). The experiments were conducted on passage 3–5 HUVECs.

### 4.5. Preparation of Dimethyl Sulfoxide (DMSO)-Induced HL-60 (D-HL-60) Cells

HL-60 cells, which are a human promyelocytic leukemia cell line, were acquired from the Bioresource Collection and Research Center (Hsinchu, Taiwan). They were maintained in suspension culture in RPMI 1640 medium, supplemented with 20% heat-inactivated FBS (fetal bovine serum) and 2 mM L-glutamine and antibiotics, in a humidified 5% CO_2_ incubator. Viability was quantified by Trypan Blue dye exclusion. Treatment of HL-60 cells with DMSO induces granulocytic differentiation [30]. To induce differentiation, viable log-phase cells were resuspended in complete RPMI 1640 growth medium containing 1.5% DMSO and were incubated for 7 days. After 7 days of 1.5% DMSO treatment, cells with distinct lobular nuclei or banded nuclei and cytoplasmic granules were present, both of which are hallmarks of granulocyte differentiation (Supplementary Figure S1a). We studied differentiation in D-HL-60 cells by monitoring the expression of CD11b, which is a well-established surface marker for differentiated neutrophils [31]. Flow cytometry was used to confirm the very high expression of CD11b in D-HL-60 cells (68%, Supplementary Figure S1b).

### 4.6. Flow Cytometry

To examine surface CD11b expression, HL-60 cells after the treatment with and without DMSO were stained with APC-Cy™7 Mouse Anti-Human CD11b (557754, BD, San Diego, CA, USA). The cells were then subjected to flow cytometric analysis with a FACS Canto II (BD Biosciences, San Jose, CA, USA). Cells were analyzed using a gating strategy. An initial gate to isolate neutrophils was established based on the characteristics of FSC and SSC. CD11b expression of 10,000 gated neutrophils was determined as CD11b^+^ cells. Data were analyzed by FlowJo software (Tree Star, Ashland, OR, USA) with 10,000 events per sample.

### 4.7. Western Blotting

Harvested homogenized mouse lung tissues and HUVECs (6 × 10^5^ per well) were placed in an RIPA lysis buffer prepared with a freshly added protease inhibitor cocktail (1862209, Thermo Fisher, Waltham, MA, USA) and 0.1 M Na_3_VO_4_. The samples were subsequently centrifuged at 20,000 rpm for 10 min at 4 °C and stored at −20 °C. Equal amounts of each protein homogenate were resolved on 7.5–10% sodium dodecyl sulfate-polyacrylamide gel electrophoresis (SDS-PAGE) gels and transferred onto polyvinylidene fluoride (PVDF) membranes. After the membranes were blocked in Tris-buffered saline with Tween-20 (TBS-T) and 5% milk, they were incubated with primary antibodies recognizing β-actin (1:5000, 20536-1-A, Proteintech, Rosemont, IL, USA), VCAM-1 (1:100, #14694, Cell Signaling, Denver, CO, USA), ICAM-1 (1:100, ab171123, Abcam, Cambridge, UK), VLA-4 (1:1000, 19676-1-AP, Proteintech, Rosemont, IL, USA), and LFA-1 (1:1000, Proteintech, Rosemont, IL, USA). After 1 h at room temperature (RT), the blots were washed in TBS-T and incubated in appropriate horseradish peroxidase-conjugated secondary antibodies. Enhanced chemiluminescence (ECL) reagents (WBKLS0500, 1:1, Millipore, Burlington, MA, USA) were added to visualize the bound antibodies. To quantify protein levels, the blots were exposed to film, and densitometric readings of the immunoreactive bands were obtained by using ImageJ software.

### 4.8. Histology and IHC

Lung tissue from the iPSC and control groups was excised 24 h after LPS-induced lung injury. Tissue was fixed in 4% paraformaldehyde for 10 min, embedded in paraffin, and sectioned for staining with primary antibodies recognizing VCAM-1 (1:100, #14694, Cell Signaling, Denver, CO, USA), ICAM-1 (1:100, ab171123, Abcam, Cambridge, UK), VLA-4 (1:100, ab202969, Abcam, Cambridge, UK), and LFA-1 (1:1000, Proteintech, Rosemont, IL, USA) with Envision^®^ + Dual Link System-HRP (DAB^+^) kits (K4065, DAKO, Carpinteria, CA, USA). In brief, the sections were deparaffinized with xylene, dehydrated with ethanol, and heated in 0.01 M citrate buffer (pH 6.0). Endogenous peroxidase activity was deactivated in 3% H_2_O_2_ for 10 min at room temperature. The sections were blocked with a blocking buffer (K4065 kit). Secondary anti-rabbit, antibody-coated polymer peroxidase complexes (K4065 kit) were then applied for 30 min at room temperature, followed by treatment with substrate/chromogen (K4065 kit), and further incubation for 5–15 sec at room temperature. After counterstaining the slides with hematoxylin (109249, MERCK, Darmstadt, Germany) for 10 sec, the sections were washed in running water for 10 min. The sections were observed and photographed with an Olympus AX80 fluorescence microscope (Olympus America, Melville, NY, USA). The percentage of IHC signal per photographed field was analyzed with Image process software (Image-Pro Plus, Media Cybernetics, Inc., Silver Spring, MD, USA).

### 4.9. Immunofluorescence Stain

The sections were deparaffinized with xylene, dehydrated with ethanol, and heated in 0.01 M citrate buffer (pH 6.0). After the sections were blocked with 3% FBS (in PBS) for 60 min at room temperature, VCAM-1 (1:100, #14694, Cell Signaling, Denver, CO, USA) and VLA-4 (1:100, ab202969, Abcam, Cambridge, UK) were applied overnight at 4 °C. The next day, goat anti-rabbit IgG H&L (Alexa Fluor^®^ 488) (1:400, ab150077, Abcam, Cambridge, UK) was incubated as a secondary antibody at 37 °C for 2 h. The slides were mounted using mounting medium with DAPI (H-1200, Vector Laboratories, CA, USA) to obtain nuclear staining. Images of the cells were taken on a Fluoview confocal microscope (FV10i, OLYMPUS, Tokyo, Japan).

### 4.10. Scoring of Lung Injuries

To quantify the severity of ALI by histology, a lung injury score was assessed for each mouse. Two investigators independently evaluated each hematoxylin and eosin (H&E)-stained slide in a blinded manner. Three-hundred alveoli were counted on each slide at 400× magnification, and a score was assigned according to previously established criteria [5,6]. Lung injury score = [(alveolar hemorrhage points/no. of fields) + 2 × (alveolar infiltrate points/no. of fields) + 3 × (fibrin points/no. of fields) + (alveolar septal congestion/no. of fields)]/total number of alveoli counted.

### 4.11. In Vitro D-HL-60 Cells–Endothelial Cell Adhesion Assay

HUVEC were seeded in a 3 cm diameter dish and covered 95% of the bottom (Supplementary Figure S2). HUVECs were stimulated with LPS (10 μg/mL) for 4 h and were treated with hiPSC-CM (1:4 to HUVEC culture medium, or were co-cultured with hiPSC or hFs (1:1 to HUVEC) for 2 h. Neutrophil-like D-HL-60 cells were stained with 4 μg/mL Calcein-AM (#354216, Corning, Corning, NY, USA) for 1 h. After washing twice with PBS, D-HL-60 cells were co-cultured with HUVEC at 37 °C for 2 h. Subsequently, all liquid was removed and washed twice with 1× PBS. Fluorescent cells were then counted after being fixed with 4% paraformaldehyde [32].

### 4.12. In Vitro D-HL-60 Cells Transendothelial Migration (TEM) Assay

The TEM ability of D-HL-60 cells was determined by the Transwells technique (BD Biosciences, San Jose, CA USA) (Supplementary Figure S2). HUVECs were seeded in the upper compartment of 3.0-µm 24-well Transwells at 1 × 10^5^ cells per well for 8 h. After 8 h, the medium was replaced with fresh medium, and 1 × 10^5^ D-HL-60 cells per well were added. A pre-test of neutrophil TEM assay showed that the addition of 10 μg/mL LPS for 2 h induced adequate neutrophil TEM responses (Supplementary Figure S3). The culture medium in the upper well contained 10 μg/mL LPS. D-HL-60 cells were stimulated with LPS and were treated with hiPSC-CM (1:4 to HUVEC culture medium) or were co-cultured with hiPSCs or hFs (1:1 to HUVEC) for 2 h. The culture medium with 1 ng/mL recombinant human MIP-2 (Peprotech, Rocky Hill, NJ, USA) was placed in the lower well as a chemotactic stimulus. After incubation for 2 h, the cells remaining on the upper surface of the membranes were removed by gently wiping it with a cotton swab. D-HL-60 cells that migrated through the membranes were stained with DAPI and were visualized with an inverted fluorescence microscope (Olympus, Tokyo, Japan), with five random microscopic fields being counted per well [33].

### 4.13. Human Angiogenesis Factors Protein Array

To evaluate the concentrations of angiogenic factors in all culture groups, supernatants acquired after 2 h in the culture were screened using the human angiogenesis array (QAH-ANG-1000, RayBiotech, Norcross, GA, USA). Protein expression levels were quantified by measuring the standard curve from a serial dilution standard in this kit.

### 4.14. Statistical Analysis

Data are presented as the mean ± standard error of the mean (SEM) or standard deviation (SD) for each experimental group. One-way analysis of variance (ANOVA) and the Tukey–Kramer multiple comparisons test or the pair-wise Student’s *t*-test were used. *p*-values of less than 0.05 were considered significant. Statistical analysis was performed utilizing SPSS software (Version 17.0, SPSS, Inc., Chicago, IL, USA).

## Data Availability

Data are available on request from the authors.

## Supplementary Materials

The following are available online at https://www.mdpi.com/article/10.3390/ijms22115554/s1, Figure S1: Treatment with dimethyl sulfoxide (DMSO) induced granulocytic differentiation in HL-60 cells, a human promyelocytic leukemia cell line, Figure S2: The diagrams for the cell adhesion assay and transendothelial migration assay, Figure S3: Pre-test of neutrophil TEM assay showed that administering 10 μg /mL LPS for 2 h incubation in D-HL-60 cell/HUVECs transmigration assay had a good response on neutrophil TEM.

## Abbreviations

Acute lung injury (ALI); acute respiratory distress syndrome (ARDS); induced pluripotent stem cells (iPSCs); conditioned medium of induced pluripotent stem cells (iPSC-CM); mesenchymal stem cells (MSCs); lipopolysaccharide (LPS); acute lung injury (ALI); murine iPSCs (miPSCs); human iPSC (hiPSC); murine iPSC-CM (miPSC-CM); human iPSC-CM (hiPSC-CM); mouse embryonic fibroblasts (MEFs); human skin fibroblasts (hFs); dimethyl sulfoxide (DMSO); DMSO-induced HL-60 cells (D-HL-60 cells); human umbilical vein endothelial cells (HUVECs); polymorphonuclear neutrophils (PMNs); nuclear factor (NF)-κB; intercellular adhesion molecule-1 (ICAM-1); vascular cell adhesion molecule-1 (VCAM-1); very late antigen-4 (VLA-4); lymphocyte-function-associated antigen-1 (LFA-1); leukemia inhibitory factor (LIF); transendothelial migration (TEM); institutional animal care and use committee (IACUC); phosphate-buffered saline (PBS); bronchoalveolar lavage fluid (BALF); immunohistochemistry (IHC); ethylenediaminetetraacetic acid (EDTA); sodium dodecyl sulfate-polyacrylamide gel electrophoresis (SDS-PAGE); polyvinylidene fluoride (PVDF); Tris-buffered saline with Tween-20 (TBS-T); room temperature (RT); enhanced chemiluminescence (ECL); hematoxylin and eosin (H&E); standard error of the mean (SEM); standard deviation (SD); analysis of variance (ANOVA).
