# Supplementary material for: Induced Pluripotent Stem Cell-Derived Conditioned Medium Promotes Endogenous Leukemia Inhibitory Factor to Attenuate Endotoxin-Induced Acute Lung Injury
Source: Int J Mol Sci. 2021 May 24;22(11):5554. doi: 10.3390/ijms22115554 (PMC8197417; doi:10.3390/ijms22115554)
Supplement: Supplementary file 1 [file ijms-22-05554-s001.zip › ijms-1229663-supplementary.pdf]

International Journal of
Molecular Sciences

MDPI

Supplemental Figure 1a

(A) (B)

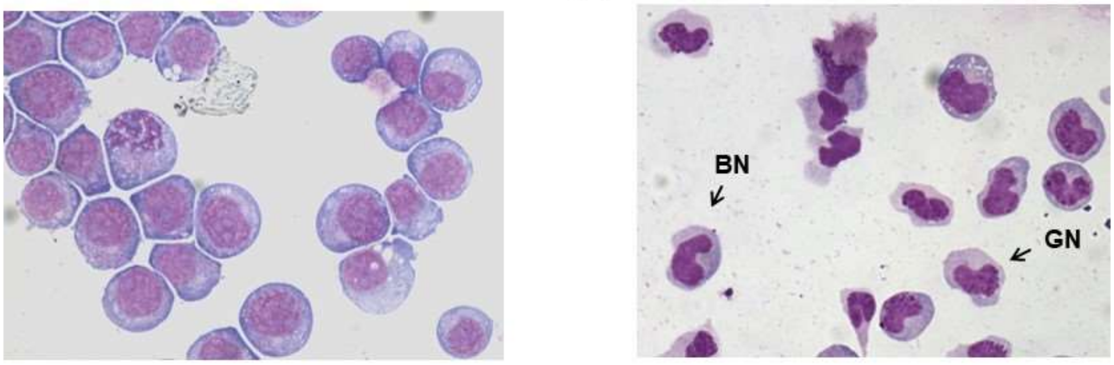

HL-60 HL-60 with DMSO 1.5%, 7 days

Supplemental Figure 1b

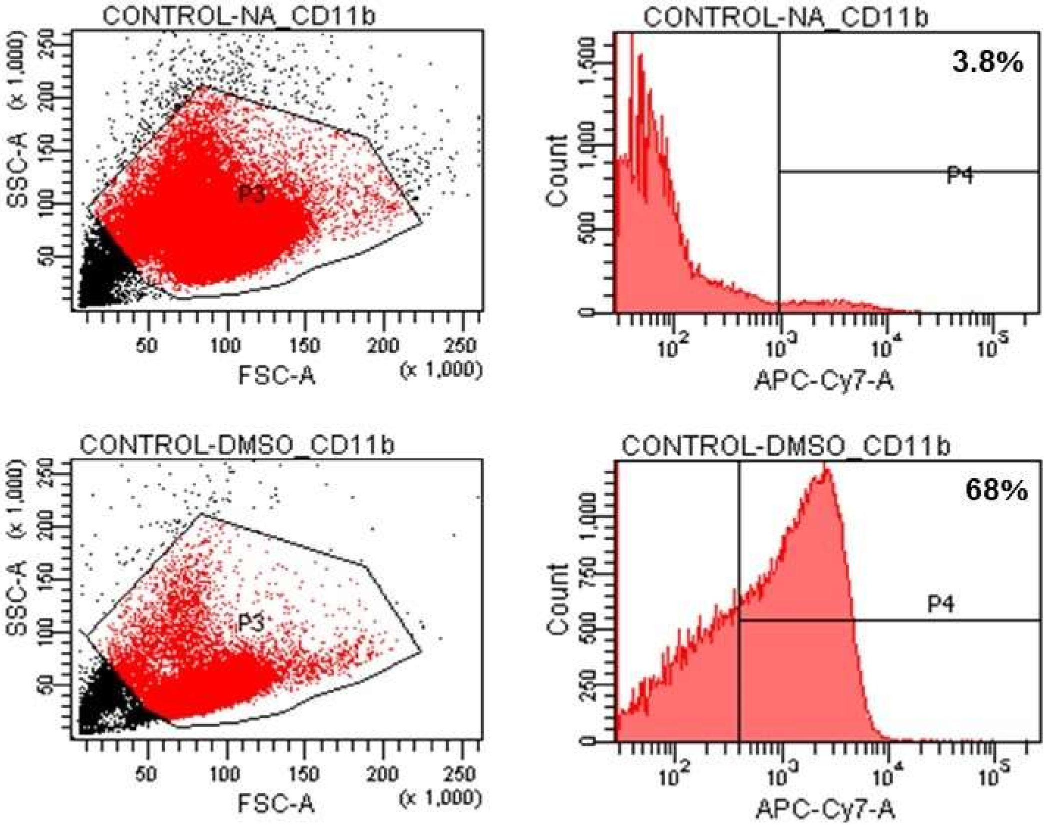

Figure S1. Treatment with dimethyl sulfoxide (DMSO) induced granulocytic differentiation in HL-60 cells, a human promyelocytic leukemia cell line. **(a)** Morphological changes to HL-60 cells after treatment with DMSO. Seg-mented neutrophil-like cells were observed after 7 days of 1.5% DMSO treatment. **(b)** Expression of CD11b in HL-60 cells significantly elevated from 3.8% to 68% after treatment with DMSO.

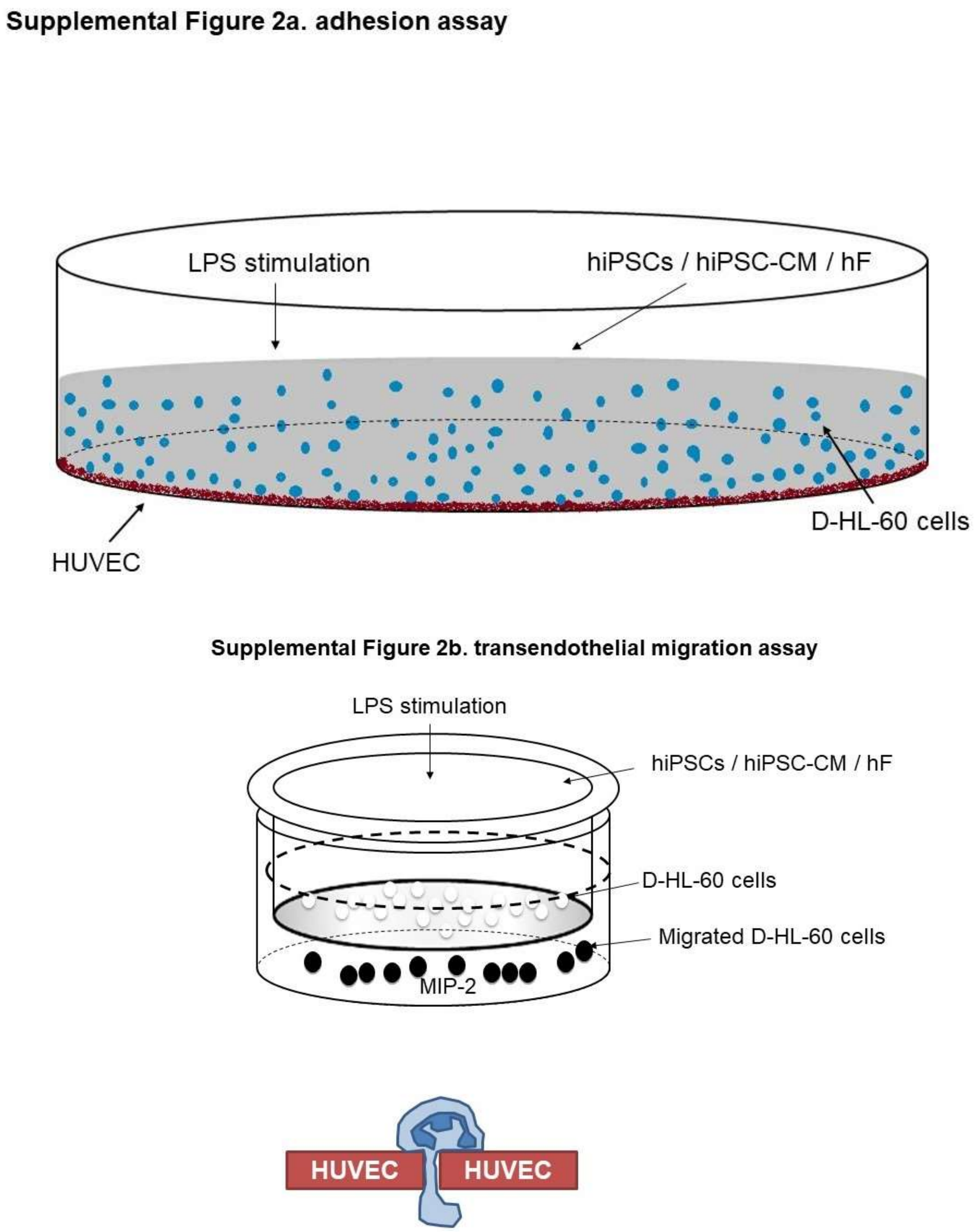

Figure S2. The diagrams for the cell adhesion assay and transendothelial migration assay.

Supplemental Figure 3a

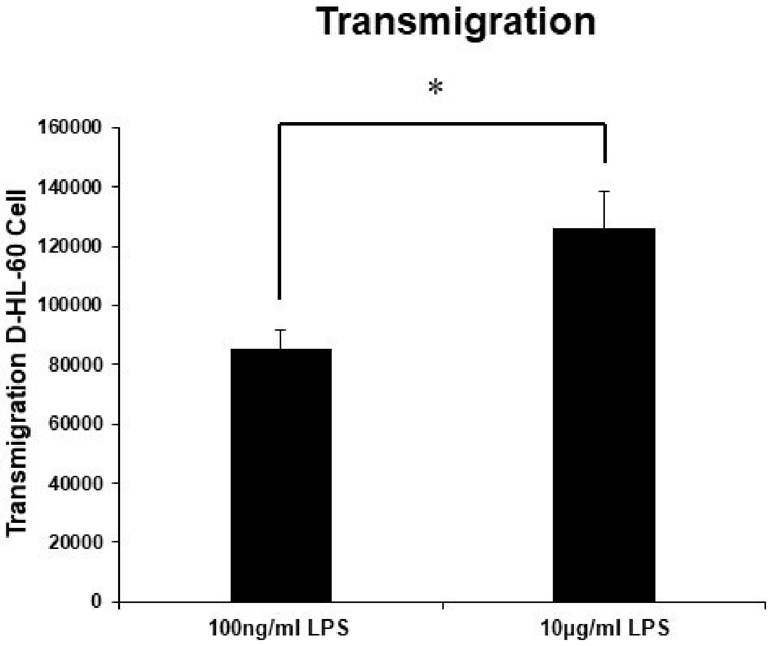

Supplemental Figure 3b

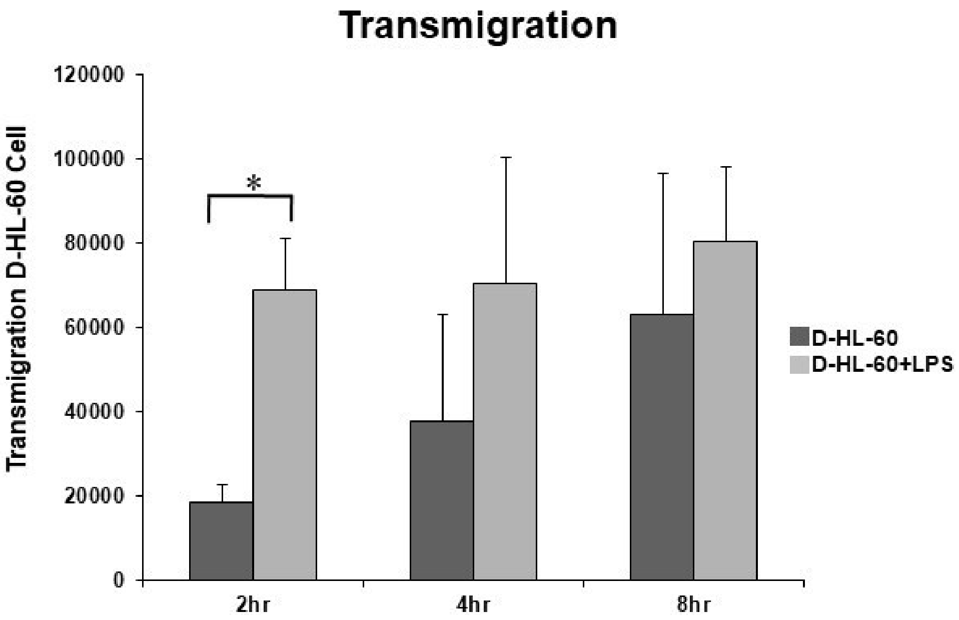

Figure S3. Pre-test of neutrophil TEM assay showed that administering 10 μg /mL LPS for 2 h incubation in D-HL-60. cell/HUVECs transmigration assay had a good response on neutrophil TEM. **(a)** *In vitro* neutrophil TEM assay, 10 μg / mL LPS induced more TEM of neutrophils compared to 100 ng/mL LPS. **(B)** *In vitro* neutrophil TEM assay; 2 h

incubation revealed significant differences between D-HL-60 cells and D-HL-60 cells after administering 10 μg /mL LPS. *$P < 0.05$; n = 6 per group.
